# Gallium-68 perfusion positron emission tomography/computed tomography to assess pulmonary function in lung cancer patients undergoing surgery

**DOI:** 10.1186/s40644-016-0081-5

**Published:** 2016-08-20

**Authors:** Pierre-Yves Le Roux, Tracy L. Leong, Stephen A Barnett, Rodney J. Hicks, Jason Callahan, Peter Eu, Renee Manser, Michael S. Hofman

**Affiliations:** 1 Division of Radiation Oncology and Cancer Imaging, Peter MacCallum Cancer Centre, St. Andrews Place, East Melbourne, VIC 3002 Australia; 2 Nuclear Medicine department, Brest University Hospital, EA3878 (GETBO) IFR 148, Brest, France; 3 The University of Melbourne, Parkville, Australia; 4 Department of Surgery, Austin Health, Heidelberg, Australia; 5 Department of Surgery, Royal Melbourne Hospital and Peter MacCallum Cancer Centre, St. Andrews Place, East Melbourne, VIC 3002 Australia; 6 Department of Cancer Medicine, Peter MacCallum Cancer Centre, St. Andrews Place, East Melbourne, VIC 3002 Australia; 7 Department of Respiratory Medicine, Royal Melbourne Hospital, Grattan Street, Parkville, VIC Australia; 8 Service de médecine nucléaire, CHRU de Brest, 29609 Brest Cedex, France

**Keywords:** PET/CT, Perfusion, Gallium-68, Surgery, Lung cancer

## Abstract

**Background:**

Pre-operative evaluation of lung cancer patients relies on calculation of predicted post-operative (PPO) lung function based on split lung function testing. Pulmonary perfusion (Q) PET/CT can now be performed by substituting Technetium-99 m labeling of macroaggregated albumin (MAA) with Gallium-68. This study compares Q PET/CT with current recommended methods of pre-operative lung function assessment.

**Methods:**

Twenty-two patients planned for curative surgical resection (mean FEV1 77 %, SD 21 %; mean DLCO 66 %, SD 17 % predicted) underwent pre-operative Q PET/CT. Sixteen patients also underwent conventional lung scintigraphy. Lobar and lung split PPO lung function were calculated using Q PET/CT and current recommended methods, i.e. calculation based on anatomical segments for lobar function, and conventional perfusion scan for pneumonectomy. Bland-Altman statistics were used to calculate agreement between methods for PPO FEV1 and PPO DLCO.

**Results:**

While mean split lobar functions were comparable, there was variation on an individual level between Q PET/CT and the anatomical method, with absolute difference over 5 % and 10 % in 37 % and 11 % of patients, respectively. For lobectomy the mean difference in PPO FEV1 was−1.2, but limits of agreement were−10 to 8.1 %. For DLCO, values were−1.1 % and−9.7 to 7.5 %, respectively. For pneumonectomy, PPO FEV1 values were−0.4 and−5.9 to 5.1 %. For DLCO, values were 0.3 % and−5.1 to 4.6 %.

**Conclusions:**

While anatomic estimation provides “fixed” results, split lobar functions computed with Q PET/CT vary widely, reflecting the intra and inter-individual variability of regional lung function. Further studies to assess the role of Q PET/CT in predicting peri-operative risk in lung cancer patients planned for lobectomy are warranted.

## Background

Lung cancer is the leading cause of cancer-related mortality worldwide [1]. In individuals with stage I and II non-small cell lung cancer, surgery is the treatment of choice if the tumour is considered resectable and the patient is considered fit for surgery [2]. Despite refinements in lung resection techniques, post-operative morbidity and mortality are significant and only 20–25 % of patients will eventually undergo surgery [3]. Most patients with lung cancer are former or current smokers, which increases operative risk, particularly due to chronic obstructive pulmonary disease (COPD). The dilemma then arises of whether to perform potentially life saving surgery in a patient who has an increased risk of operative mortality or of significant post-operative dyspnoea. Therefore, accurate evaluation of pre-operative lung function is imperative to estimate the risk of both short and long-term post-operative adverse events, and to select patients who will derive maximum benefit with minimal risk from surgery [3].

Current guidelines recommend a series of investigations to risk stratify patients [4–6]. Initial tests include measurement of the forced expiratory volume in 1 second (FEV1) and diffusion capacity of the lung for carbon monoxide (DLCO). However, these tests alone are limited because they do not take into account the high degree of inter-patient variability including regional heterogeneity of any underlying lung disease, the extent of lung to be resected, and the contribution of the portion of lung to be removed to overall lung function. Accordingly, split lung function testing is used in conjunction with FEV1 and DLCO to assess the functional contribution of the lung to be resected and to calculate a predicted post-operative (PPO) value of lung function. The American College of Chest Physicians (ACCP) guidelines recommend that both FEV1 and DLCO be measured and that both PPO FEV1 and PPO DLCO are calculated in all patients with lung cancer being considered for surgery [6]. To compute PPO values, guidelines recommend the use of conventional perfusion scintigraphy using macroaggregated albumin (MAA) labelled with Technetium-99 m (^99m^Tc) before pneumonectomy or an anatomic method based on counting the number of functional segments to be removed before lobectomy [6].

Our group has demonstrated the feasibility of transitioning from conventional single photon techniques to positron emission tomography (PET) technology for lung scintigraphy [7]. ^99m^Tc can be substituted by Gallium-68 (^68^Ga), a positron-emitting radionuclide, to label MAA, which are trapped in the lung capillaries so that local concentration is related to the regional pulmonary blood flow. The regional distribution of perfusion within the lungs is then possible using PET technology. This offers a unique opportunity to improve the diagnostic performance of lung perfusion imaging, due to the higher sensitivity, spatial resolution, speed of acquisition and, most importantly, quantitative capability of PET in comparison to conventional scintigraphy [8]. In a recent study, we showed a high degree of correlation between ventilation-perfusion PET/CT functional lungs volumes and pulmonary function tests (PFTs) parameters [9], suggesting significant potential in management of patients with pulmonary disease. We have also explored this technology for individualising radiotherapy treatment plans [10, 11] and assessing radiation injury to lungs [10, 12].

In order to assess the potential utility of this new technology for pre-operative assessment of lung cancer patients, we aimed to compare the information provided by perfusion (Q) PET/CT imaging with that of current recommended methods, when assessing split lung function and computing PPO values of lung function in lung cancer patients being considered for surgery.

## Methods

### Patients

In this retrospective series, patients referred to our PET centre for Q PET/CT imaging as part of their preoperative evaluation for lung surgery by a single surgeon were identified between 2013 and 2014. Patients with biopsy proven lung malignancy were planned for surgery with curative intent based on exclusion of distant metastatic disease by FDG PET/CT. Patients with clinically suspected lung malignancy were planned for intraoperative frozen section to confirm presumed malignancy prior to resection with curative intent. All patients underwent PFTs and gated perfusion PET/CT as part of pre-treatment evaluation. 16 patients also underwent planar lung scintigraphy using ^99m^Tc-MAA. Three patients were ‘salvage cases’ ie they had undergone chemo radiation with curative intent months prior and had failed locally. Out of the 22 patients, 13 underwent a lobectomy, 4 a segmentomy, 1 a pneumonectomy and 4 patients did not have curative surgery, respectively. The study was performed in accordance with the Declaration of Helsinki and was approved by the institutional ethics committee (Number 13/152).

### Pulmonary function tests

Spirometry was performed in accordance with the joint European Respiratory Society (ERS) and American Thoracic Society (ATS) clinical practice guidelines [13, 14]. Post bronchodilator FEV1 was expressed as an absolute value and a percentage of predicted and DLCO as a percentage of predicted.

### Q PET/CT protocol

All patients underwent a respiratory-gated (4D) PET-CT lung perfusion scan using a procedure that we have previously described [15]. Patients were imaged on a GE Discovery 690 PET/CT scanner (GE Medical Systems Milwaukee, WI, USA) after injection of approximately 50 MBq of ^68^Ga-MAA [7]. The perfusion PET was acquired as a two-bed acquisition encompassing the apex to base of both lungs planned by a scout CT. Each bed position was acquired for 5 min.

### Split lung function calculation

#### Conventional methods

As a reference standard, lobar and lung split function were computed according to current recommended methods, using an anatomic estimation before lobectomy and by a conventional planar perfusion scan before pneumonectomy [6].

For lobar split function assessment, the following equation was used:

$$ Relative\  function = Number\  of\  function al\  or\  unobstructed\  lung\  segments\  in\  the\  lobe\  of\  in terest\ /\  total\  number\  of\  function al\  or\  unobstructed\  lung\  segments. $$

The total number of segments for both lungs was 19 (10 in the right lung: 3 in the upper, 2 in the middle, 5 in the lower; and 9 in the left lung: 3 in the upper division, 2 in the lingula and 4 in the lower) [16].

For lung split function assessment, conventional planar perfusion scintigraphy was used as follow:

$$ \mathrm{Relative}\ \mathrm{function} = \mathrm{perfusion}\ \mathrm{in}\ \mathrm{the}\ \mathrm{lung}\ \mathrm{of}\ \mathrm{in}\mathrm{terest}\ /\ \mathrm{total}\ \mathrm{lung}\ \mathrm{perfusion} $$

### Q PET/CT

Lobes were delineated on CT images using MIMimage analysis software (MIM 5.4.4; MIMSoftware, Cleveland, OH, USA). In the left upper lobe, the lingula and the left upper division were delineated. Segmentation was subsequently applied to the PET images and the following equation was used to compute split lung function for each lobe and lung:

$$ Relative\  function = perfusion\  in\  the\  region\  of\  in terest\ /\  total\  lung\  perfusion $$

### PPO lung function calculation

PPO FEV1 as an absolute value (in litres) and a percent predicted, and PPO DLCO as a percent predicted, were computed for all 22 patients for 2 scenarios-1 assuming each would undergo pneumonectomy for the index lesion and for this scenario we compared Q PET/CT with planar VQ (in accordance with current guidelines) and in the second scenario we assumed each patient would undergo lobectomy for the index lesion and compared Q PET/CT with the anatomical method for calculating post op lung function (as is recommended in current guidelines).

PPO values were calculated using the following equations:

$$ \begin{array}{l}PPO\ FEV1 = pre- operative\ FEV1\ x\  fraction\  of\  total\  function\ to\  be\  removed\\ {}PPO\  DLCO = pre- operative\  DLCO\ x\  fraction\  of\  total\  function\ to\  be\  removed\end{array} $$

### Statistical analysis

All statistical tests were carried out using GraphPad Prism 5 (La Jolla, CA, USA). The mean, standard deviation, and ranges were computed for all split lung function and PPO PFTs values. Limits of agreement between PPO PFTs values were analysed by means of Bland-Altman analysis.

## Results and discussion

Twenty-two patients (11 males, 11 females; mean age 67 years, range 38–82 years) were analysed. The mean pre-operative FEV1 was 2.04 L (SD: 0.65 L; range: 1.00–3.26 L) and the mean pre-operative percentage of predicted FEV1 was 77 % (SD: 21 %; range: 33–123 %). The mean pre-operative percentage of predicted DLCO was 66 % (SD: 17 %; range: 39–110 %). Primary lesion was located in the right upper lobe in 9 patients, the right middle lobe in 2, the right lower lobe in 4, the left upper division in 6, and the left lower lobe in 1 patient.

### Split lung function

**Fig. 1:**
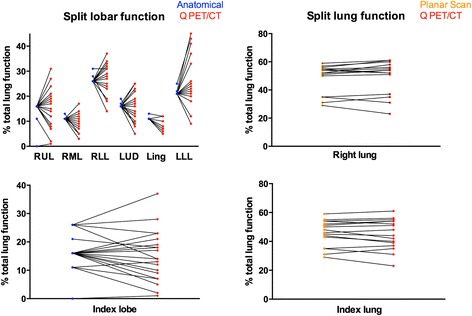
Paired split lung function using Q PET/CT and the recommended methods, i.e. the anatomical method for split lobar function, and planar scintigraphy for relative lung function

**Fig. 2:**
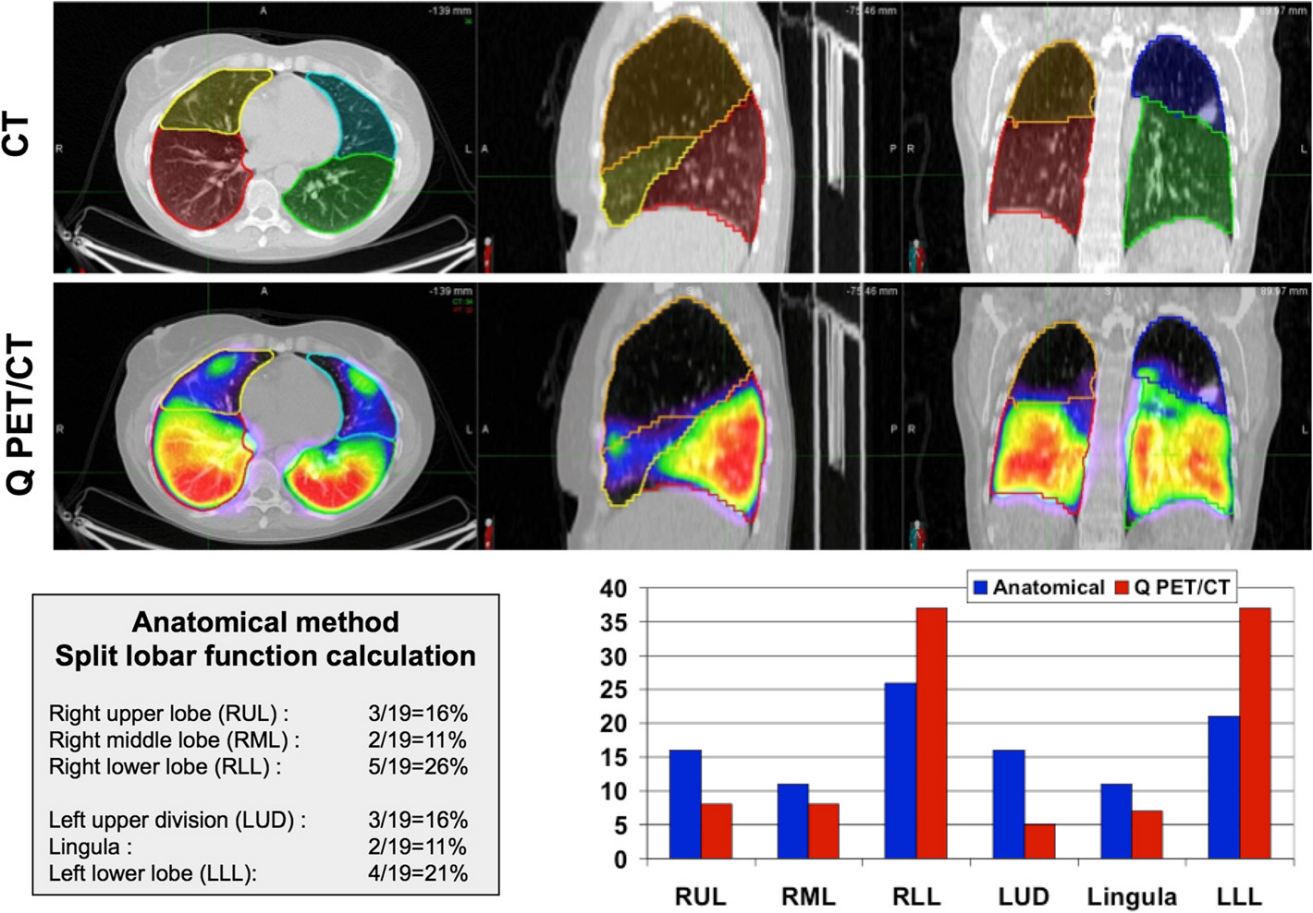
Example of discordant lobar split function distribution

Mean of split lung functions computed with Q PET/CT and the reference methods were not statically different. However, standard deviation and range of split lobar function were much wider using Q PET/CT rather than the anatomical method (See Table 1 and Fig. 1). The absolute difference in lobar split function between Q PET/CT and the anatomic method was greater than 5 % of total lung function in 49 of 132 lobes (37 %), and greater than 10 % in 15 of 132 of lobes (11.4 %). The absolute difference in lung split function between Q PET/CT and planar perfusion scan was greater than 5 % of total lung function in 19 % of lungs but never more than 10 %. Figure 2 shows an example of different lobar split function using Q PET/CT or the anatomic estimation.

**Table 1:** Comparison of split function computed with Q PET/CT and the recommended methods (anatomic estimation for lobar split function, conventional perfusion scan for lung split function) for all lobes and lungs regardless of index lesion

	Anatomic estimation			Q PET/CT		
	Mean	sd	Range	Mean	sd	Range
RUL	15	3	0–16	15	8	1–31
RML	11	0	11–13	9	4	3–17
RLL	27	1	26–31	27	6	14–37
LUD	16	1	16–19	16	6	5–25
Lingula	11	0	11–13	7	2	5–12
LLL	21	1	21–25	26	11	9–48
Index lobe	17	6	0–26	15	7	1–37
	Planar perfusion scan			Q PET/CT		
	Mean	sd	Range	Mean	sd	Range
RL	49	10	29–59	51	11	23–54
LL	51	10	41–71	49	11	36–77
Index lung	46	9	29–59	45	11	23–61

Results are expressed as percentage of total lung function

### PPO lung function after lobectomy

**Fig. 3:**
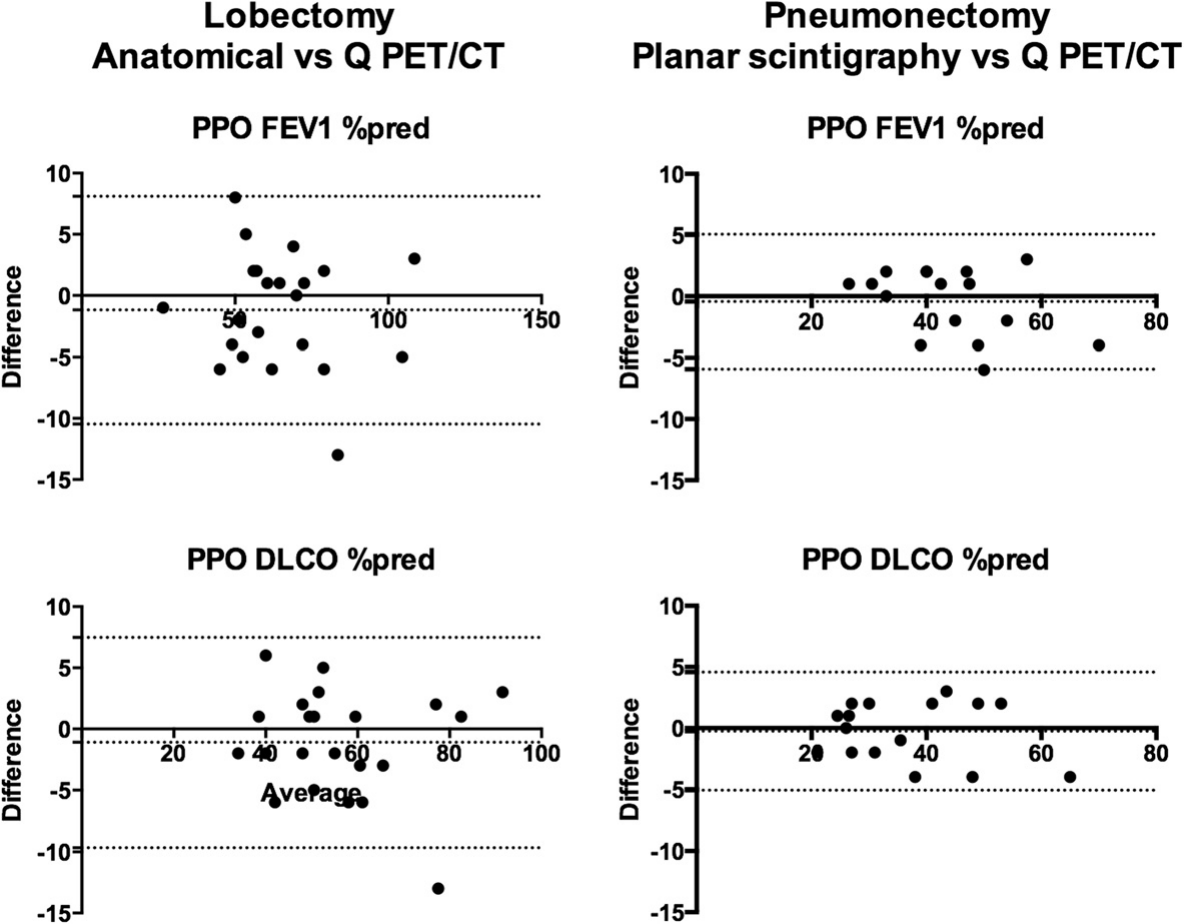
Bland-Altman plot of PPO FEV1 and DLCO as percent predicted, after lobectomy or pneumonectomy. The bias and the limits of agreement between PPO values are displayed in each graphics

The mean, standard deviation and range of Predicted post-operative % predicted FEV1, absolute FEV1 and DLCO, as calculated by the anatomical method or Q PET/CT are presented in Table 2. The mean of the difference and the limits of agreement between PPO as % predicted FEV1 and % predicted DLCO are shown in Fig. 3.

**Table 2:** PPO PFTs values after lobectomy of the index lobe

	Anatomical			Q PET/CT		
	Mean	sd	Range	Mean	sd	Range
FEV1 % pred	64	19	26–110	65	19	27–107
FEV1 L	1.69	0.52	0.79–2.65	1.72	0.54	0.82–2.81
DLCO % pred	55	15	33–93	56	15	35–90

### PPO lung function after pneumonectomy

The mean, standard deviation and range of PPO % predicted FEV1, FEV1 and % predicted DLCO using the planar scintigraphy or Q PET/CT are presented in Table 3. The mean of the difference and the limits of agreement between PPO as % predicted FEV1 and % predicted DLCO are shown in Fig. 3.

**Table 3:** PPO PFTs values after pneumonectomy of the index lung

	Planar			Q PET/CT		
	Mean	sd	Range	Mean	sd	Range
FEV1 % pred	44	11	27–68	44	12	26–72
FEV1 L	1.10	0.41	0.58–2.12	1.12	0.46	0.59–2.25
DLCO % pred	37	12	20–63	37	13	22–67

## Discussion

In the present study, we found that lobar lung split functions calculated by Q PET/CT differ from that estimated with the current recommended methodology [6]. While anatomic estimation provides “fixed” results, relative lobar functions vary widely using Q PET/CT, consistent with known inter-individual variability of regional lung function. The impact of Q PET/CT when calculating PPO lung function was less marked, but not trivial clinically, in this small series including patients without significant lung function impairment. On the other hand, Q PET/CT does not seem to provide major differences as compared with conventional planar scintigraphy in patients being assessed for pneumonectomy. This is not entirely unexpected since both reflect the ratio of perfusion to the left and right lungs.

We compared lobar split function computed with Q PET/CT and the current recommended method, i.e. an anatomical approach based on counting the number of functional segments to be removed [6]. While mean lobar split functions were very close with both methods, standard deviations and ranges were much wider using Q PET/CT, with lobar relative function being either higher or lower than that predicted with the anatomic method. Overall, absolute difference in lobar split function between both methods was greater than 5 % of total lung function in 37.1 % lobes and greater than 10 % in 11.4 % of lobes. Given the heterogeneity of regional lung function, especially in patients with COPD [17], these results are likely to be more representative of the inter-individual variability of regional lung function as compared with the fairly “fixed” results provided by the anatomical method.

The question then arises regarding the impact of such results when estimating the risk of adverse surgical events, i.e. when calculating PPO FEV1 and DLCO. In the present study, the limits of agreement between PPO values of lung function predicted from Q PET/CT and the anatomical method were up to 10 % (PPO FEV1:−10–8.1 % 1; PPO DLCO:−9.7–7.5 %). These limits of agreement appear large and clinically significant in the setting of a pre-operative workup of a lung cancer patients being considered for surgery. In a study of 1,428 subjects undergoing lung resection, Alam et al. found a 10 % increase in the risk of complications for every 5 % decrement in PPO lung function [18]. In the ACCP guidelines for physiologic evaluation of lung cancer patients before surgery, a functional algorithm based on measurement of PPO FEV1 and PPO DLCO in all patients is proposed. In particular, a cut-off value of 60 % is proposed for both PPO FEV1 and PPO DLCO to select patients with a low risk for surgery. If one parameter is lower than 60 %, further testing is recommended. In our series of 22 patients, 3 would have had a different management using Q PET/CT rather than the anatomical method when computing PPO values. In addition, using the historical 40 % cut-off to select patients suitable for surgery with an acceptable clinical outcome, decision-making would have been different in 2 additional patients. Nevertheless, there were no patients with PPO FEV1 or PPO DLCO lower than 30 %, which has been recently described as a more adapted cut-off [6, 19].

When simulating a pneumonectomy of the index lung, the impact of transitioning from conventional planar scintigraphy to PET technology was less convincing. Mean, standard deviation and graphical analysis of split lung functions demonstrate close results with both methods. Limits of agreement were smaller (PPO FEV:−5.9–5.1 % 1; PPO DLCO:−5.1–4.6 %) as compared with a lobectomy. However, both lung and lobar split lung function calculation may be advantageous in patients with borderline lung function to compare the post-operative risk of both pneumonectomy and lobectomy to aide surgical decision making in the event that intraoperative findings preclude safe oncological lobectomy. In that setting, Q PET/CT may offer the advantage of providing an accurate assessment of the risk of both types of surgery in a single test.

Perfusion PET/CT imaging represents a promising alternative to current methods owing to several advantages. The acquisition time is around six minutes less than for conventional planar scintigraphy using a dual-detector gamma camera but provides fully tomographic images of higher resolution. SPECT/CT can provide tomographic images but has a significantly longer acquisition time and provides lower resolution. As with conventional scintigraphy, there are no known contraindications or acute side effects (allergy) associated with the radiotracers. The effective radiation dose of the scan is low, approximately 1 mSv for the PET acquisition plus an additional 1–2 mSv for the low dose CT component. In addition, the number of particles typically used for a fresh ^68^Ga MAA administration is approximately half of that used for a fresh ^99m^ Tc-MAA administration, which may be an advantage of VQ PET as compared with VQ SPECT in patients with pulmonary hypertension. In our institution, there is no significant increase in cost or processing as regards Ga68-MAA labelling but this may vary according to local expertise and facilities. Finally, ^68^Ga is produced by an on-site generator enabling on-demand availability similar to ^99m^Tc, but with a longer shelf-life of 9–12 months versus 1–2 weeks for ^99m^Tc generator. The ^68^Ga generator is increasingly available owing to its use for neuroendocrine [20] and prostate cancer imaging. With PET/CT and ^68^Ga becoming increasingly available, we envisage that widespread adoption of V/Q PET/CT could become a reality as part of a more general more of diagnostic nuclear medicine towards PET/CT technology [8].

This study has several limitations. First, PPO lung functions were not correlated with actual post-operative lung function. Nevertheless, there is evidence than PPO values are prognostic factors for short and long-term postoperative risk rather than accurate predictors of post-operative lung function [21]. In particular, many studies have reported an improvement in pulmonary function after lung surgery in some patients with COPD in keeping with a lung volume reduction effect [22]. On the other hand, the risk of post-operative complications has been linked to PPO lung function [18]. In this small series, we did not assess the prognostic value of PPO PFT values computed using Q PET/CT. As a first necessary step, we showed that this new technology provides different information as compared with current recommended methods, which justify, from our point of view, further studies whose objective will be to compare the prognostic value of methodologies in terms of post-operative morbidity and mortality. Second, we did not compare Q PET/CT results to other modalities such as V/Q SPECT/CT [23], quantitative CT [24] or MRI [25], which have also been proposed to predict post-operative lung function. However, we compared Q PET/CT to the currently recommended methodologies, which are likely to be the most commonly used method in clinical practice. Third, this study was limited to a small series of patients and included some patients with non-impaired lung function. Again, this was performed in keeping with clinical guidelines. Nevertheless, it is likely that the usefulness of such a modality that aims at providing a more personalized approach would be greater in patients with borderline lung function. Finally, for a variety of reasons including patient preference and logistic considerations, only approximately 1/3 of patients assessed for resection during the inclusion period underwent Q PET/CT assessment. A selection bias is therefore a possibility. However, this was a heterogeneous group of patients, likely broadly reflecting the surgeons’ standard practice.

## Conclusions

To the best of our knowledge, this study is the first assessing Q PET/CT to compute split lung function and predict PPO lung function in lung cancer surgery patients. In the pre-therapeutic work up of patients undergoing lobectomy, Q PET/CT provides different results compared to the anatomical estimation, with a wider range of results that may be more representative of the inter-individual variability of regional lung function. Further larger studies are now needed to assess if Q PET/CT allows better prediction of short and long-term outcome and may influence management of lung cancer patients undergoing surgery.

## Abbreviations

COPD, chronic obstructive pulmonary disease; DLCO, diffusion capacity of the lung for carbon monoxide; FEV1, forced expiratory volume in 1 second; MAA, macroaggregated albumin; PFTs, pulmonary function tests; PPO, Predicted post-operative; Q PET/CT, Perfusion Positron Emission Tomography/Computed Tomography; SD, Standard deviation
